# A Calcium-Dependent Chloride Current Increases Repetitive Firing in Mouse Sympathetic Neurons

**DOI:** 10.3389/fphys.2018.00508

**Published:** 2018-05-14

**Authors:** Juan Martinez-Pinna, Sergi Soriano, Eva Tudurí, Angel Nadal, Fernando de Castro

**Affiliations:** ^1^Departamento de Fisiología, Genética y Microbiología, Universidad de Alicante, Alicante, Spain; ^2^Institute of Bioengineering and CIBER de Diabetes y Enfermedades Metabólicas Asociadas, Miguel Hernández University of Elche, Elche, Spain; ^3^Instituto Cajal (CSIC), Madrid, Spain

**Keywords:** sympathetic neuron, chloride current, repetitive firing, gender differences, anthracene-9′-carboxylic acid

## Abstract

Ca^2+^-activated ion channels shape membrane excitability in response to elevations in intracellular Ca^2+^. The most extensively studied Ca^2+^-sensitive ion channels are Ca^2+^-activated K^+^ channels, whereas the physiological importance of Ca^2+^-activated Cl^-^ channels has been poorly studied. Here we show that a Ca^2+^-activated Cl^-^ currents (CaCCs) modulate repetitive firing in mouse sympathetic ganglion cells. Electrophysiological recording of mouse sympathetic neurons in an *in vitro* preparation of the superior cervical ganglion (SCG) identifies neurons with two different firing patterns in response to long depolarizing current pulses (1 s). Neurons classified as phasic (Ph) made up 67% of the cell population whilst the remainders were tonic (T). When a high frequency train of spikes was induced by intracellular current injection, SCG sympathetic neurons reached an afterpotential mainly dependent on the ratio of activation of two Ca^2+^-dependent currents: the K^+^ [I_K(Ca)_] and CaCC. When the I_K(Ca)_ was larger, an afterhyperpolarization was the predominant afterpotential but when the CaCC was larger, an afterdepolarization (ADP) was predominant. These afterpotentials can be observed after a single action potential (AP). Ph and T neurons had similar ADPs and hence, the CaCC does not seem to determine the firing pattern (Ph or T) of these neurons. However, inhibition of Ca^2+^-activated Cl^-^ channels with anthracene-9′-carboxylic acid (9AC) selectively inhibits the ADP, reducing the firing frequency and the instantaneous frequency without affecting the characteristics of single- or first-spike firing of both Ph and T neurons. Furthermore, we found that the CaCC underlying the ADP was significantly larger in SCG neurons from males than from females. Furthermore, the CaCC ANO1/TMEM16A was more strongly expressed in male than in female SCGs. Blocking ADPs with 9AC did not modify synaptic transmission in either Ph or T neurons. We conclude that the CaCC responsible for ADPs increases repetitive firing in both Ph and T neurons, and it is more relevant in male mouse sympathetic ganglion neurons.

## Introduction

As part of the autonomic nervous system, mammalian sympathetic neurons innervate and modulate the activity of several target organs, combining with the somatic nervous system to control internal homeostasis, blood pressure, and body temperature (Jänig and McLachlan, 1992; de Castro, 2016). Therefore, the correct firing of these neurons is crucial for the organism’s survival. In many respects, post-ganglionic sympathetic neurons are quite heterogeneous, for example in their anatomical location, morphology, pattern of synaptic input, neuropeptide content, passive electrical properties, modulation by neurotrophins or voltage- and Ca^2+^-dependent ion channels (de Castro, 1923, 1927, 1932, 2016; Hirst and McLachlan, 1984; McLachlan and Meckler, 1989; Gibbins, 1992; Jänig and McLachlan, 1992; Keast et al., 1993; Martinez-Pinna et al., 2000a; Li and Horn, 2006; Luther and Birren, 2009; Palus and Całka, 2016).

The spike firing of sympathetic neurons also shows some heterogeneity, and it is determined by the type, relative strength and location of their synaptic inputs, and by the biophysical properties of the cell membrane (for a review, see: McLachlan, 2003). In different *in vitro* preparations of intact sympathetic ganglia, two firing patterns in response to depolarizing current pulses have been described in different species: phasic (Ph, rapidly adapting), and tonic (T, slowly adapting: Adams and Harper, 1995; Jobling and Gibbins, 1999). In some cases, two subtypes of Ph neurons have been distinguished (Wang and McKinnon, 1995), Ph-1 neurons that fire a single potential in response to long depolarizations and Ph-2 neurons with a shorter afterhyperpolarization (AHP) that respond with 2–4 closely spaced spikes at the onset of the maintained depolarization before they undergo adaptation. This classification has since been extended to consider a third class of ganglion cells with very long AHPs that fire phasically, neurons identified in the rabbit, guinea-pig, and rat as long AHP (LAH) neurons (Cassell and McLachlan, 1987; Boyd et al., 1996). The proportion of cells from each class varies in the different ganglia and species, most neurons firing phasically in the rat superior cervical ganglion (SCG: Yarowsky and Weinreich, 1985; Wang and McKinnon, 1995). The firing pattern of mouse sympathetic neurons depends on the type of ganglia studied, SCG neurons displaying a predominant Ph firing pattern and a larger proportion of T neurons in the celiac ganglion (Jobling and Gibbins, 1999). However, recent results indicate that many sympathetic cells fire at higher frequencies and that the increase in leak current may have reduced excitability in some experiments (Springer et al., 2015), suggesting that afterdepolarizations (ADPs) in these neurons could be larger than previously thought. Firing patterns in human post-ganglionic sympathetic neurons have been studied through focal recordings, indicating that only one action potential (AP) is generated per sympathetic burst (Macefield and Elam, 2004). Furthermore, healthy women have been reported to have less sympathetic nerve activity than men (Hogarth et al., 2007).

Different ionic conductances have been used to determine firing patterns and to assess frequencies. Electrophysiological data suggest that the differences in accommodation between Ph and T sympathetic ganglion cells are due to the presence of the I_M_ K^+^ current in Ph neurons (Wang and McKinnon, 1995), which is slowly activated by depolarization (Cassell et al., 1986; Romero et al., 2004). In fact, inhibition of I_M_ by angiotensin II increases the excitability of SCG neurons (Zaika et al., 2007), whereas this current is absent (Wang and McKinnon, 1995), or at least significantly smaller (Cassell et al., 1986), in T neurons. However, it has also been proposed that the I_M_ is too small in sympathetic neurons to determine the firing pattern (Belluzzi and Sacchi, 1991). The firing frequency of T neurons is in part set by the K^+^ currents underlying AHP, particularly since its blockade with apamin enhances firing frequency (Cassell et al., 1986; Cassell and McLachlan, 1987; Wang and McKinnon, 1995; Faber and Sah, 2002). I_A_ is another K^+^ current that is significantly larger in T neurons and while it may have an important influence on firing properties (Connor and Stevens, 1971; Cassell et al., 1986), another study failed to find differences in I_A_ between Ph and T neurons (Wang and McKinnon, 1995), considering that it might only be relevant in a subset of sympathetic neurons with a small or no I_K(Ca)_. Indeed, T neurons in mice celiac ganglia were less likely to co-express both I_A_ and I_M_ than Ph neurons (Jobling and Gibbins, 1999).

Differences in inward currents might also contribute to the different firing properties of T and Ph neurons. Although inactivation of Na^+^ channels does not seem to provoke the refractoriness of Ph neurons (Wang and McKinnon, 1995), a persistent Na^+^ current was described in rat SCG neurons that allows them to oscillate in the sub-threshold range, as well as contributing to the resting membrane potential (RMP) and enhancing cellular excitability (Lamas et al., 2009). Furthermore, a voltage-dependent Cl^-^ conductance has been described in rat sympathetic neurons that control the RMP (Sacchi et al., 2003). We found that mouse SCG neurons have an inward Ca^2+^-activated Cl^-^ current (CaCC) that produces an ADP after AP firing, as the equilibrium potential for Cl^-^ ions in these cells is ≈-15 mV (de Castro et al., 1997; Martinez-Pinna et al., 2000b). Peripheral neurons and immature central neurons strongly express a Na^+^-K^+^-Cl^-^ cotransporter that mediates Cl^-^ influx (Sung et al., 2000) and thus, the Cl^-^ currents at the RMP are depolarizing (Cl^-^ ions exit the cell). By contrast, the equilibrium potential for Cl^-^ ions in the central nervous system (CNS) is much more negative (≈-70 mV) due to the K^+^/Cl^-^ cotransporter present in mature neurons (Kakazu et al., 1999). Hence, CaCC activation results in the inward flow of Cl^-^ ions, hyperpolarizing central neurons and mediating spike-frequency adaptation (Huang et al., 2012; Ha et al., 2016; Ha and Cheong, 2017).

Among the members of the anoctamin family of Cl^-^ channels, also known as the transmembrane protein (TMEM) 16 family, anoctamin 1 (ANO1, TMEM 16A) and anoctamin 2 (ANO2, TMEM 16B) are considered to be Ca^2+^-activated Cl^-^ currents (CaCCs) (Caputo et al., 2008; Yang et al., 2008). ANO1 and ANO2 are widely expressed in different tissues, and they are involved in various physiological processes. However, the distribution and activity of ANO1 and ANO2 in the brain has not been extensively studied, and it is even less clear in the peripheral nervous system (PNS). We have shown that the CaCC in SCG sympathetic neurons is activated concomitantly with the Ca^2+^-activated K^+^ current [I_K(Ca)_: de Castro et al., 1997], while it is only evident in rat SCG cells after axotomy. One feasible explanation for this is that CaCC is preferentially expressed in distal dendrites that retract following axotomy, making it possible to recording the ADP from the soma (Sánchez-Vives and Gallego, 1994). Neurons do not fire spontaneously during ADP (Sánchez-Vives and Gallego, 1994; de Castro et al., 1997; Jobling and Gibbins, 1999), yet the role of CaCCs in spike firing has not been carefully studied in sympathetic neurons. However, a Ca^2+^-activated Cl^-^ channel does contribute to the repolarization of an ADP in inferior olivary neurons and hence, it increases the firing rate in central neurons and promotes cerebellar motor learning (Zhang et al., 2017). Therefore, the balance between the activity of the inward Ca^2+^-activated Cl^-^ channels and Ca^2+^-activated K^+^ channels in mouse SCG neurons could establish adequate AP firing. Here we report that, anthracene-9′-carboxylic acid (9AC), a Cl^-^ channel blocker that blocks the CaCC in SCG cells (Baron et al., 1991; de Castro et al., 1997; Váczi et al., 2015), decreases the firing rate of mouse SCG cells. Furthermore, this CaCC was larger in male than in female mice, as was the expression of the CaCC ANO1/TMEM16A in SCG. These results ascribe a physiological role to this CaCC in controlling firing behavior in sympathetic neurons.

## Materials and Methods

### Tissue Preparation and Electrophysiology

The methods for intracellular recording have been described previously (de Castro et al., 1997; Martinez-Pinna et al., 2000b). Briefly, 8- to 13-week-old male mice (Swiss OF-1) were deeply anesthetized by an I.P. injection of sodium pentobarbitone (40 mg kg^-1^) and perfused through the heart with cold oxygenated saline until breathing stopped. Both SCG were taken from the animal and while one was pinned to the Sylgard (Dow Corning, Midland, MI, United States) bottom of a chamber (1.6 ml volume) that was continuously superfused (1.5–2.5 ml/min) with saline (mM: NaCl, 128; KCl, 5; CaCl_2_, 2.5; MgCl_2_, 1; NaH_2_CO_3_, 16; NaH_2_PO_4_, 1; glucose, 5.5) equilibrated with 95%O_2_-5%CO_2_ (pH 7.4) at room temperature (22–25°C), the other was kept in the solution at 4°C until use (no more than 6 h). All recordings were done during the first 10 h after the extraction of ganglia to prevent cellular damage. The ganglion was illuminated from one side with a fine optic fiber light source, allowing the cells on the surface to be seen using a microscope (×375–600). For the experiments shown in **Figure 5**, SCG were obtained from 8- to 13-week-old male and female mice (Swiss OF-1). All experimental procedures were performed according to the Spanish Royal Degree 1201/2005 and the European Community Council directive 2010/63/EU. The Ethics Committee from the Instituto de Neurociencias of the Universidad Miguel Hernández-CSIC (formerly Universidad de Alicante; Alicante, Spain) approved all methods used in this study (approval ID: 20147/VSC/PEA/00184). Animals were treated humanely and with care to alleviate suffering. All procedures were carried out in accordance with the approved guidelines and regulations.

Potentials were measured with respect to a Ag-AgCl pellet connected to the bath through an agar-KCl bridge. Cells were impaled with microelectrodes filled with 3 M KCl (70–90 MΩ). Data were considered only if the cell generated APs larger than 70 mV. The sampling frequency for discontinuous single-electrode current and voltage clamp was 2–6 kHz, with a duty cycle of 30/70 (Axoclamp 2B, Axon Instruments Inc.). Capacitance compensation was continuously monitored and adjusted to ensure head stage settling. Data were digitized and stored on a computer for subsequent analysis using commercial software (pCLAMP9, Axon Instruments Inc.). General parameters studied were RMP, amplitude of AP, amplitude and duration of AHPs, input resistance (R_input_), threshold potential (V_threshold_), threshold current (rheobase), amplitude of excitatory postsynaptic potentials (EPSP), and absolute potential reached at the peak of the EPSP (V_EPSP_). To generate afterpotentials, trains of 35 APs were induced by injection of short (5 ms) intracellular positive current steps at 50 Hz (de Castro et al., 1997); the values of amplitude (measured 50 ms after the end of the train, both related to the RMP, and absolute afterpotential reached) and duration of ADP were collected. Repetitive firing was evoked with 1 s depolarizing pulses of various amplitudes (0.1, 0.3, 0.5, 0.7, and 1.0 nA), and both firing frequency (number of APs generated/s) and instant frequency (first five intervals) were measured.

Tail CaCC current underlying the ADP was measured in the presence of apamin to block I_K(Ca)_ using a hybrid-clamp protocol in which APs were evoked with depolarizing current pulses and the amplifier was switched to voltage clamp mode, either after repolarization of a single AP, or 10 ms after the end of a six APs train at 40 Hz. Tail currents were recorded under voltage clamp at -55 mV after filtering at 0.3 kHz and were analyzed using commercial software (Origin, OriginLab, Northampton, MA, United States) For these experiments, neurons were impaled with microelectrodes filled with 3 M KCl (40–50 MΩ).

The preganglionic trunk was taken into a close-fitting suction electrode for stimulation, placed at 6 mm (approx.) from the ganglion. The duration of the stimulation pulses were 0.5–1 ms, and the intensity and polarity were adjusted to give a compound AP of maximum amplitude and minimum latency. The amplitude of the EPSP evoked by supramaximal stimulation (0.5–1 Hz) of the preganglionic trunk was measured during the refractory period of an intracellularly evoked AP (Purves, 1975; Gallego and Geijo, 1987; de Castro et al., 1995). The stimulus was timed so that the peak of the EPSP occurred around 10 ms after the peak of the AP.

### Solutions

The Cl^-^ channel blocker 9AC (Sigma, United States) was diluted in NaOH (1 M, and adjusted to pH 7.4 with HCl) and added to the control solution at a final concentration of 2 mM. Its effects were recorded at least 2 min after changing the solution, and the wash out times of this agent were no shorter than half an hour.

#### RNA Isolation and Quantitative Real-Time PCR (qRT-PCR) Analysis

Total RNA was extracted with an RNeasy Micro Kit (Qiagen, Germany) according to the manufacturer’s instructions, and quantified with Nanodrop 2000 (Thermo Scientific, United States). RNA was reverse-transcribed using a High Capacity cDNA Reverse Transcription kit (Applied Biosystems, United States). Amplifications were performed using SYBR Green Supermix (Bio-Rad, United States) in a CFX96^TM^ Real-time PCR System (Bio-Rad, United States) following the manufacturer’s instructions and the following primer sequences (5′-3′): TAACCCTGCCACCGTCTTCT and AA GCCTGTGAGGTCCCATCG for *Ano1* (slope = -3.2109, *R*^2^ = 0.998, efficiency = 104.85%) (Wu et al., 2014), and GGTTAAGCAGTACAGCCCCA and TCCAACACTTCGAGAGGTCC for the housekeeping gene *Hprt* (slope = -3.3168, *R*^2^ = 0.998, efficiency = 100.21%). The resulting values were analyzed with CFX Manager Version 1.6 (Bio-Rad, United States), and relative values were calculated with the Pfaffl method (Pfaffl, 2001).

### Statistical Analysis

All values are expressed as the mean ± standard error of mean (SEM). Statistical analyses were performed using SigmaStat (Jandel Scientific, Germany) employing a Student’s *t*-test, a Mann–Whitney rank sum test, a paired *t*-test and a Pearson correlation test as appropriate. The threshold of statistical significance was *P* < 0.05 for all comparisons.

## Results

### Firing Patterns and Afterpotentials in Mouse Superior Cervical Ganglion Cells

**FIGURE 1:**
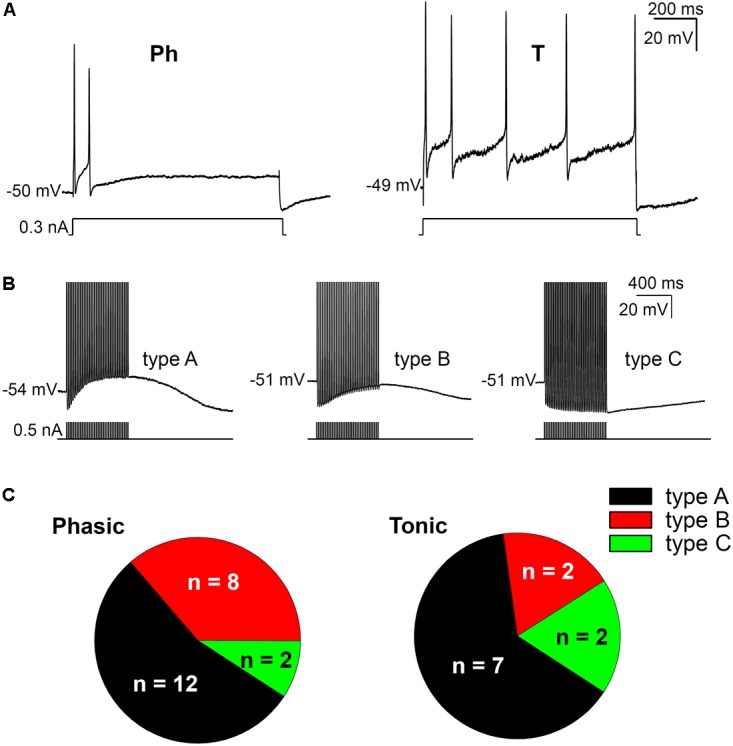
Firing patterns and afterpotentials of mouse sympathetic neurons. **(A)** Phasic (Ph) firing behavior of a cell in response to a depolarizing pulse (0.3 nA, 1 s) and cell showing a tonic (T) response to a similar depolarization. In both cases, pulse amplitude was less than 1.5 times the rheobase. The resting membrane potential (RMP) of cells is indicated at the beginning of each recording. Calibration bars apply to both panels (as in **B**). **(B)** Classification of cells according to their afterpotentials: with clear ADP after the train (type A); with a certain degree of CaCC activation, although the concomitant activation of I_K(Ca)_ results in a negative afterpotential 50 ms after the end of the train, but more positive than the AHP of the first spike (type B); finally, with a clear AHP after the train, similar to those of the individual spikes (type C). Action potentials (APs) were evoked with short (5 ms) intracellular current pulses at 35 Hz shown in each recording below the membrane potential. Spikes are truncated at the top. **(C)** Differential distribution of the three neuron types showed in **B** in both Ph and T SCG neurons.

The firing pattern of SCG ganglion cells was determined by applying depolarizing 1 s pulses of threshold and twice threshold current intensities. In accordance with previous studies (Cassell et al., 1986; Wang and McKinnon, 1995; Jobling and Gibbins, 1999), the cells that stopped firing within the first 500 ms were classified as phasic (Ph; **Figure 1A**) and those that fired beyond this time were classified as tonic (T; **Figure 1A**). In terms of Ph neurons, those that elicited a single spike upon depolarization were designated as Ph-1 and those that responded with a few closely spaced APs were designated as Ph-2 (Wang and McKinnon, 1995). As no significant differences in electrical properties were observed between Ph-1 and Ph-2 neurons (data not shown), to simplify the results the Ph-1 and Ph-2 neurons will be considered together in a single group (Ph). We detected a mixed firing pattern in mouse SCG cells in which CaCCs were present (**Figure 1C**) and of 33 cells studied, 22 (67%) were considered as Ph (**Figures 1A,C**) and 11 (33%) showed T firing (**Figures 1A,C**). Only one of the cells could be considered as LAH, although the duration of the AHP following a single AP was considerably shorter (hundreds of milliseconds) than that described for this third class of cells (several seconds: Cassell and McLachlan, 1987). Given that this was only one cell and in light of the differences with LAH cells in other species as a specific kind of phasic firing cell, we included it in the Ph group to simplify the description of the results.

When SCG neurons were challenged with a train of 35 APs through the injection of positive intracellular current at 50 Hz steps to generate afterpotentials, a predominantly ADP (type A, **Figure 1B**), a partially activated ADP (type B, **Figure 1B**) or a predominant AHP (type C, **Figure 1B**) was recorded after the train of spikes. Hence, the degree of activation of Ca^2+^-dependent conductances [i.e., CaCCs and I_K(Ca)_] varies among cells, although the CaCC was predominant, as type 1 (predominant ADP) was the afterpotential best represented in Ph and T neurons (**Figure 1C**). The fact that the three types of afterpotentials were recorded in both Ph and T neurons suggests that that the Cl^-^ current underling the ADPs is not responsible for determining the firing pattern of mouse sympathetic cells. Further evidence that ADP does not favor establishing a tonic pattern is the fact that no differences were observed in the amplitude of the ADP between Ph and T neurons, which was either related to RMP or the absolute after-potential reached 50 ms after the application of the train of spikes (**Table 1**).

**Table 1:** Electrophysiological properties of phasic and tonic mouse sympathetic neurons.

	Phasic (*n* = 22)	Tonic (*n* = 11)
RMP (mV)	-54 ± 1.6	-54 ± 2.3
Amplitude of action potential (mV)	91 ± 4.8	87 ± 3.0
Amplitude of AHP post-spike (mV)	-14 ± 1.7	-17 ± 1.2
Duration of AHP post-spike (ms)	360 ± 41	371 ± 45
R_input_ (Mω)	75 ± 19 (*n* = 6)	85 ± 12 (*n* = 6)
V_threshold_ (mV)	-58 ± 2.7	-59 ± 3.4
Rheobase (nA)	0.21 ± 0.02; **	0.11 ± 0.02; **
First spike latency pulse (ms)	8.7 ± 0.7	8.1 ± 0.6
Amplitude of ADP post-train (mV)	1.2 ± 2.6	1.2 ± 4.6
After-potential post-train (mV)	-52 ± 3.2	-52 ± 4.6
Firing freq. pulse 0.5 nA (spikes/s)	2.1 ± 0.2; **	6.9 ± 0.8; **
Firing freq. pulse 1.0 nA (spikes/s)	5.1 ± 0.7; **	10.7 ± 1.9; **

*^∗∗^P < 0.01 (Student’s *t*-test).*

**FIGURE 2:**
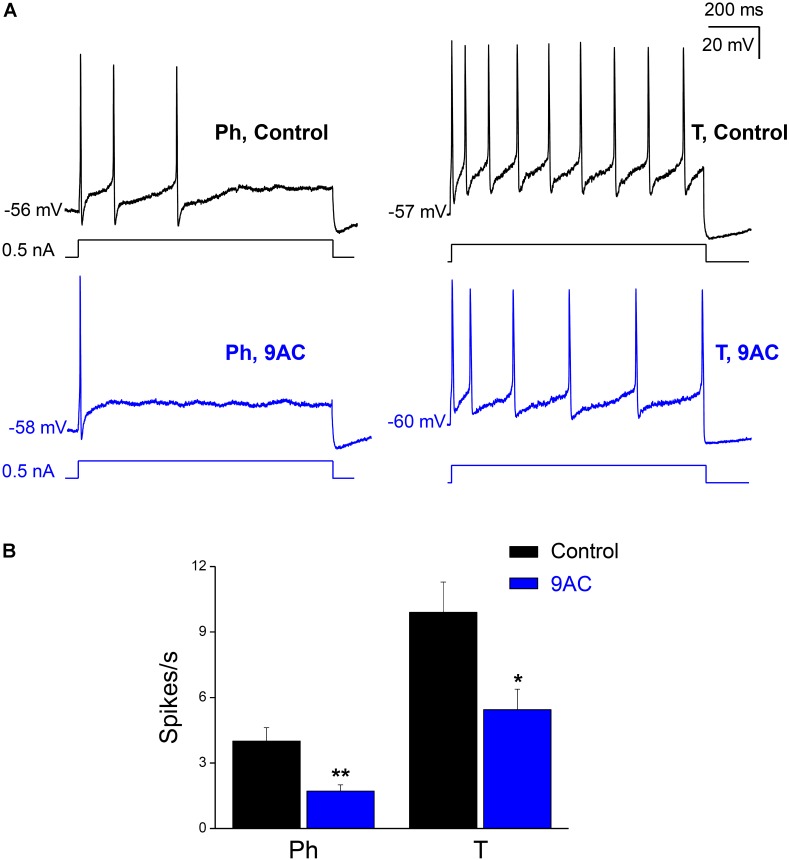
The chloride channel blocker 9AC diminishes firing frequency in both Ph and T neurons. **(A)** Repetitive firing of a Ph (left) neuron in control solution after the injection of 0.5 nA depolarizing current (black upper traces) and its modification after the addition of 2 mM 9AC (blue lower traces). The same effect in a T neuron (right). The injected current protocol is shown in each recording below the membrane potential. The RMP of cells is indicated at the beginning of each recording. Calibration bars apply to both panels. **(B)** Significant reduction of firing frequency elicited with 1.0 nA depolarizations in the presence of 2 mM 9AC for both populations of Ph (*n* = 9) and T sympathetic neurons (*n* = 9). Paired *t*-test: ^∗^*P* < 0.05; ^∗∗^*P* < 0.01.

Interestingly, even with smaller depolarizations, repetitive firing cells were observed (**Figures 1A**, **2A**), and 13 of the 22 Ph neurons elicited more than one spike with 0.5 nA depolarizations, which differs from previous observations in the rat and mouse SCG neurons (Wang and McKinnon, 1995; Jobling and Gibbins, 1999). This was also observed in T and Ph neurons with a more hyperpolarized RMP than the average (≈-65 mV; not shown). Moreover, and in contrast to previous observations (Wang and McKinnon, 1995; Jobling and Gibbins, 1999), the frequency increased with the amplitude of the depolarizing pulse (see below), although there was no significant change in spike height during the prolonged depolarization (1 s) induced by the current step (**Figures 1A**, **2A**). These observations confirm that Ph and T firing patterns coexist in mouse sympathetic ganglion cells. All these results, and the ones in the next sections, were obtained from male SCG neurons, with the exception of the results shown in **Figure 5** in which male and female were compared.

### Electrophysiological Properties of Phasic and Tonic Mouse SCG Cells

No differences between Ph and T neurons were found for either the RMP or for the characteristics of single APs (**Table 1**), and no differences were observed between the input membrane resistance (R_input_) of Ph and T sympathetic neurons (**Table 1**). However, a significantly smaller rheobase was measured in T neurons (**Table 1**), although no differences were observed when either the threshold membrane potential for firing APs (V_threshold_) or the latency of the first spike evoked (measured at peak) were examined (**Table 1**) across the range of depolarizations studied. Similar to our data, the rheobase in the rat is 0.05–0.2 nA, and there are no differences between the Ph SCG neurons and other sympathetic neurons with T firing properties (Wang and McKinnon, 1995). Nevertheless, cells exhibiting a T response to depolarization obviously had a higher firing frequency than Ph ones (**Table 1**; *P* < 0.01, Student’s *t*-test for both 0.5 and 1.0 nA pulses).

### Blocking ADP Reduces the Firing Rate

We tested how blocking the CaCC with 9AC (2 mM) affected the firing response of both Ph and T sympathetic ganglion cells. Both the V_threshold_ (-43 ± 2.7 mV) and rheobase (0.16 ± 0.02 nA) did not change when recordings were performed in the presence of 9AC (-44 ± 3.7 mV and 0.21 ± 0.03 nA, respectively). Accordingly, the latency of the first spike evoked (measured at peak) did not differ significantly between the controls (8.6 ± 0.63 ms for 0.5 nA depolarizing pulses, 5.7 ± 0.49 ms for pulses of 1.0 nA) and in the presence of 9AC (11.6 ± 2.39 ms and 5.7 ± 0.44 ms, respectively: equivalent results were obtained when the other pulses were studied). These results suggest that CaCC does not fulfill an important role in the generation of the first AP, consistent with the lack of effect of 9AC on single AP properties (see below).

When we tested the effects of 9AC on the firing frequency of Ph and T neurons separately (**Figure 2A**), there was a significant reduction in the number of spikes per second in the presence of 9AC in both cases (**Figure 2B**). Hence, 9AC affects all the cells similarly, allowing us to use the entire population of Ph and T neurons (in total, *n* = 18) to assess the effects of 9AC on repetitive firing.

**FIGURE 3:**
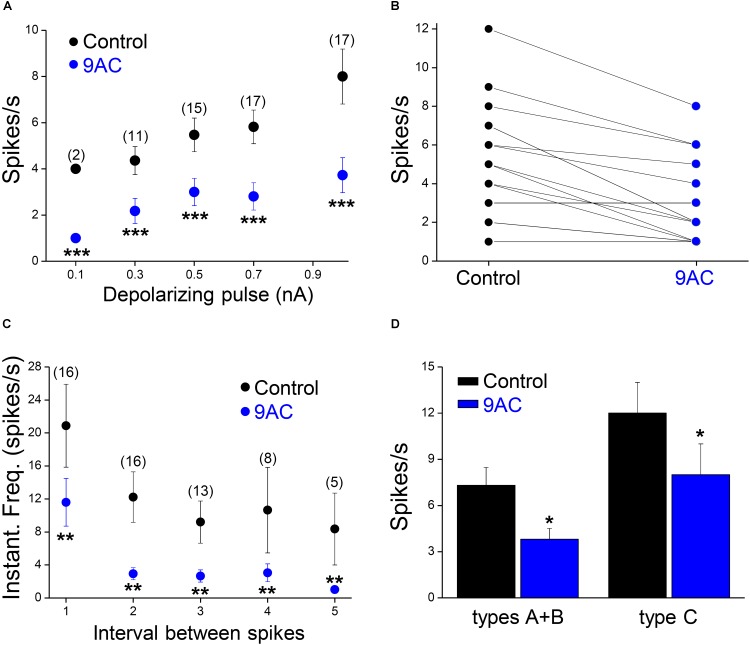
The chloride channel blocker 9AC reduces firing frequency and instantaneous frequency in almost all sympathetic neurons. **(A)** The reduction in firing frequency was observed for all the amplitudes of the depolarizing pulse used (black circles for control solution and blue circles for 2 mM 9AC solution). Only cells firing two or more spikes in the control were considered (numbers in brackets). **(B)** The number of APs elicited is larger in control than in 9AC in the majority of cases (15 out of 18) with 0.5 nA depolarizing pulses. **(C)** The instantaneous frequency of spikes was reduced for all of the five first intervals between consecutive spikes studied with depolarizations of 1.0 nA. Symbols are the same as in **A**. The number of cells studied in each case is shown in brackets. **(D)** Effects of CaCC blockade with 9AC on firing frequency separately in cells with (types A and B) and without (type C) ADP for 1.0 nA depolarizing pulses. Paired *t*-test: ^∗∗^*P* < 0.01; ^∗∗∗^*P* < 0.001 **(A,C)** and Student’s *t*-test: ^∗^*P* < 0.05 **(D)**.

While one half of the cases studied in control conditions did not fire a single spike with depolarizing pulses of 0.1 nA, all of them generated at least one spike when 0.3 nA pulses (less than double the intensity of the rheobase) were applied. To study the effect of 9AC on repetitive firing, we focused on those neurons that elicited two or more spikes after depolarization in the control solution (**Figure 2A**). The number of cells that underwent repetitive firing in the control solution increased with the amplitude of the pulse (**Figure 3A**: number of cells firing two or more APs in brackets). The number of spikes decreased significantly in the presence of 9AC across the whole range of depolarizations tested (**Figure 3A**), as evident when the spike frequency of each neuron before and after 9AC application was assessed (**Figure 3B**). The spike frequency was reduced in 15 out of 18 cells and in the other 3 cells it was unchanged, in no cells did it increase. As mentioned above, no change in spike height was observed during the pulse, either in control or in the presence of 9AC. The instantaneous firing frequency also diminished in the presence of 9AC. Indeed, when the first five intervals between consecutive APs were studied for both 1.0 (**Figure 3C**) and 0.5 nA (data not shown), there were significant differences for the first and all the other intervals (**Figure 3C**).

Of the 18 cells studied in the presence and absence of 9AC solutions, 13 were type A cells and the other 3 were type B accordingly to the ADPs (see **Figure 1B** for description of the afterpotential types), although in 2 cells CaCCs were apparently no activated at all (type C). Irrespective of the activation of CaCCs, the firing rate was significantly reduced (*P* < 0.01, paired *t*-test) in the presence of 9AC (**Figure 3D**). Interestingly, two type C neurons showed a T firing pattern, whereas all the Ph neurons displayed clear or partial activation of CaCCs (types A or B). This is consistent with the possibility that CaCCs are present in all sympathetic ganglion cells, including those that appear not to develop ADPs due to the distal dendritic location of the Ca^2+^-activated Cl^-^ channels (de Castro et al., 1997; Jobling and Gibbins, 1999).

These results reveal that blocking CaCCs with 9AC produces the same general effect in all sympathetic neurons, affecting repetitive firing but not the first spike generated by long depolarizing pulses. These effects were fully reversed by a 30 min washout of the drug (see section “Materials and Methods”).

### 9AC Blocks After-Depolarization

**FIGURE 4:**
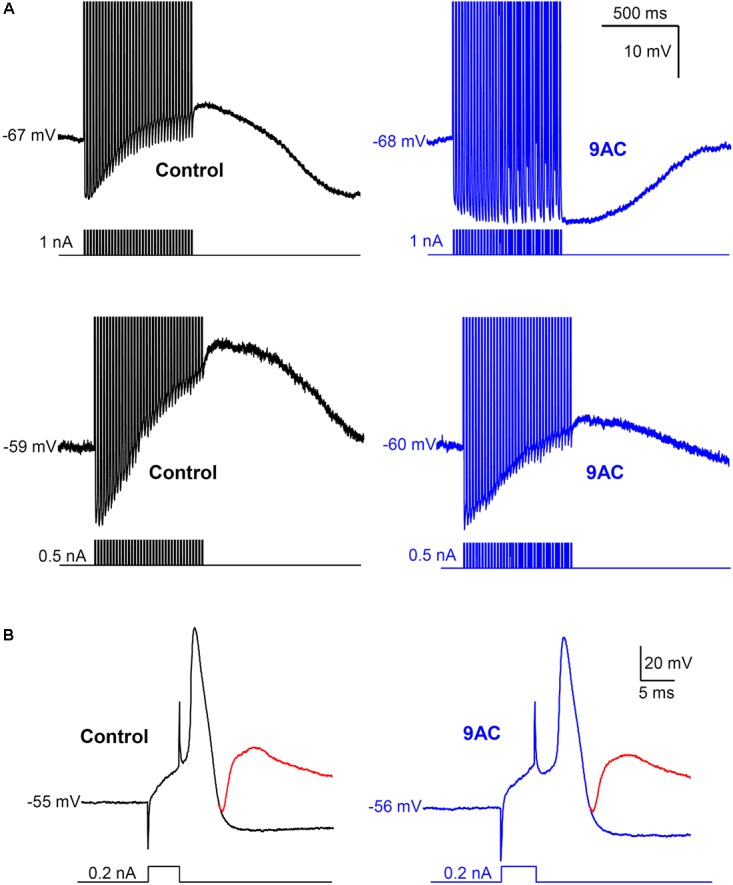
Anthracene-9′-carboxylic acid (9AC) abolishes ADPs without affecting synaptic transmission. **(A)** Representative recordings of ADPs were evoked after a train of 35 APs in control solution (black traces). ADPs disappeared completely (top panel) or were significantly reduced (low panel) after the addition of 2 mM 9AC (blue traces). Recordings of top panels are from the same cell before and after application of 9AC. The same applies to low panels. APs were evoked with short (5 ms) intracellular current pulses at 35 Hz shown in each recording below the membrane potential. Spikes are truncated at the top. Calibration bars apply to all panels. **(B)** Representative recording of an excitatory post-synaptic potential (EPSP) elicited with preganglionic stimulus in the refractory period of the AP of one cell in control solution. Addition of the chloride channel blocker 2 mM 9AC does not significantly modify the amplitude of EPSP. Two recordings are overimposed in **B**, one without stimulation of the preganglionic trunk (black and blue traces for control and 9AC, respectively) and the other with its stimulation and the evoked EPSP (red traces). Calibration bars apply to both panels. APs were evoked with a short (5 ms) intracellular current pulse shown in each recording below the membrane potential. The RMP of the cells is indicated at the beginning of each recording.

As reported previously (de Castro et al., 1997; Martinez-Pinna et al., 2000b), application of 9AC (2 mM) markedly decreased the amplitude and duration of the ADP (**Figure 4** and **Table 2**). Other electrophysiological parameters studied were not affected by 9AC, including the rheobase and V_threshold_ (see above), although there was a mild hyperpolarization of the cells (**Table 2**). Similarly, the properties of individual APs were not affected by 9AC (**Table 2**). When we compared the cells that hyperpolarized by ≥3 mV in the presence of 9AC (*n* = 6) with those that experienced smaller hyperpolarizations or mild depolarizations (*n* = 12), the effects of 9AC on firing frequency and instantaneous frequency were again identical (data not shown). Similarly, the hyperpolarization of 3–5 mV in these cells seemed to produce no marked effects in control conditions (see below).

**Table 2:** Anthracene-9′-carboxylic acid (9AC) affects only ADP.

	Control (*n* = 18)	9AC (*n* = 18)
RMP (mV)	-56 ± 1.9	-60 ± 2.0; *
Amplitude of action potential (mV)	90 ± 1.9	91 ± 1.9
Half-duration of action potential (ms)	2.11 ± 0.14	2.10 ± 0.15
Amplitude of AHP post-spike (mV)	-14 ± 0.9	-15 ± 0.9
Duration of AHP post-spike (ms)	372 ± 26	446 ± 37
R_input_ (MΩ)	77 ± 10.7	97 ± 10.4
Amplitude of ADP post-train (mV)	11 ± 2.5	-3 ± 2.5; **
Duration of ADP post-train (ms); *⇓*	942 ± 148	422 ± 103; *

*^∗∗^*P* < 0.01, ^∗^*P* < 0.05 different from control (paired *t*-test). *⇓:* only cells with remaining ADP in 9AC were included (*n* = 4)*.

Interestingly, the AHP that normally follows AP firing in sympathetic neurons is not affected by 9AC (**Table 2**), in the presence or absence of ADP (type A and B or type C neurons, respectively), indicating that the principal current responsible for AHP, the I_K(Ca)_, is not modified by this Cl^-^ channel blocker. This is also consistent with the fact that clear AHP follows either a train of spikes (**Figure 4A** top panels) or the partially reduced ADP generated by the train (**Figure 4A** low panels, see below). No effects were observed when the NaOH in the 9AC vehicle was added alone to the control solution (see section “Materials and Methods”). Furthermore, the recording time did not affect any of the electrical properties (see Supplementary Figure S1).

### 9AC Does Not Affect the Amplitude of the Excitatory Post-synaptic Potentials

To study the role of ADP in synaptic transmission, the amplitude of excitatory post-synaptic potentials (EPSPs) was measured in the refractory period of an intracellularly evoked spike in 22 neurons (**Figure 4B**). This population was composed of 4 type C cells (with AHPs) and 18 that produced ADPs after the train of spikes (types A and B), and the firing pattern was also recorded in some cells, 9 Ph and 4 T neurons. Given that no differences were observed when cells with ADPs or AHPs, or Ph and T neurons were considered separately (data not shown), we considered these as a single population when studying the effect of 9AC on synaptic transmission. The EPSP amplitude did not change in the presence of AC (control 23 ± 1.4 mV vs. 9AC 21 ± 1.4 mV, *n* = 22: **Figure 4B**), even 40 min after exposure to 9AC. The same result was observed for the absolute V_EPSP_ (control -45 ± 4.7 mV vs. 9AC -45 ± 4.0 mV, *n* = 22). Together, these results suggest that Ca^2+^-activated Cl^-^ channels do not participate in synaptic transmission in mouse SCG cells.

### The Ca^2+^-Activated Cl^-^ Currents Are Larger in Male Than in Female Mice

**FIGURE 5:**
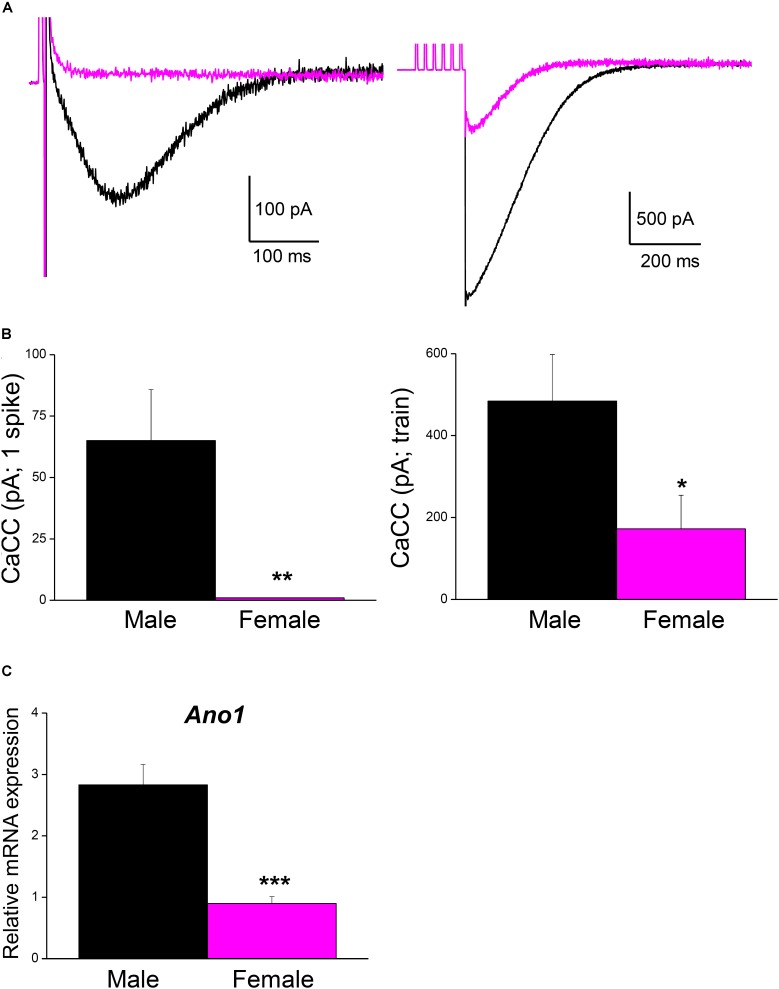
Ca^2+^-activated Cl^-^ current is larger in male than in female mouse SCG neurons. **(A)** Representative recordings of isolated CaCC after a single AP (left) and after a train of 6 spikes at 40 Hz (right) in an hybrid-clamp protocol in the presence of apamin to block I_K(Ca)_ (see section “Material and Methods”). APs were evoked with short (5 ms) intracellular current pulses (0.5–1 nA). Recordings from male (black) and female (magenta) are superimposed. **(B)** Average values of isolated CaCCs after a single AP (left) and after a train of 6 spikes at 40 Hz (right) in males (8 cells; black) and females (8 cells; magenta). Student’s *t*-test: ^∗^*P* < 0.05; ^∗∗^*P* < 0.01. **(C)** SCG mRNA expression levels of *Ano1* (male *n* = 9, female *n* = 8), normalized to that of *Hprt*. Mann–Whitney test, ^∗∗∗^*P* < 0.0001.

Male mice were used in all the experiments indicated above. However, the fact that the firing frequency of SCG neurons in our study was higher than that reported in mice (Jobling and Gibbins, 1999) and other species (Wang and McKinnon, 1995; Boyd et al., 1996), where mixed populations of adult SCG neurons from animals of both sexes were used, which prompted us to investigate the magnitude of CaCCs in female mice as well. Interestingly, the CaCCs underlying the ADP in neurons from males (recorded in an hybrid-clamp protocol, see section “Materials and Methods”) was considerably larger than in neurons from female mice, both after a single spike (**Figures 5A,B**; left panels) and after a train of six spikes at 40 Hz (**Figures 5A,B**; right panels). In these experiments, apamin was employed to block the I_K(Ca)_ in order to measure CaCCs in isolation (de Castro et al., 1997; Martinez-Pinna et al., 2000b). The larger CaCCs in male mice may therefore be explained by the increased firing rate reported here. To assess whether this difference was due to the distinct expression of CaCCs in male and female SCG, we measured the mRNA transcripts encoding the anoctamin 1 (ANO1, TMEM 16A) CaCCs by RT-qPCR. Accordingly, we found that ANO1 was more strongly expressed in male than in female neurons (**Figure 5C**).

## Discussion

The aim of this work was to elucidate a possible role of CaCCs in modifying the firing properties of mouse sympathetic neurons. This Ca^2+^-dependent Cl^-^ current is present in normal conditions in these cells and it is responsible for inducing an ADP when activated by the entry of Ca^2+^ associated with the firing of APs (de Castro et al., 1997; Jobling and Gibbins, 1999; Martinez-Pinna et al., 2000b). We had previously shown that the current underlying ADP is a Ca^2+^-dependent current, as the ADP is completely abolished by extracellular application of Cd^2+^ or in Ca^2+^-free conditions (Sánchez-Vives and Gallego, 1994; de Castro et al., 1997). Furthermore, the current underlying ADP is a Cl^-^ current as the substitution of external NaCl with isethionate or sucrose shifts the reversal potential of ADP.

Many of the agents that block Cl^-^ channels, like 4,4′-Diisothiocyano-2,2′-stilbenedisulfonic acid (DIDS) and niflumic acid, are not specific to CaCCs and they also have an effect on Ca^2+^ currents (Reinsprecht et al., 1995). This lack of specificity makes them inappropriate to study CaCCs. Nonetheless, 9AC was previously reported to block CaCCs and consequently, the ADP resulting from CaCC activation (de Castro et al., 1997; Martinez-Pinna et al., 2000b), without affecting Na^+^, K^+^, or Ca^2+^ currents (Martinez-Pinna et al., 2000b; Váczi et al., 2015). A voltage-dependent inward Cl^-^ current that is active in the subthreshold range of membrane potential has been detected in rat sympathetic neurons, and it was blocked by 9AC (Sacchi et al., 1999). It is likely that such a current, if present in mice SCG neurons, could contribute to the effects of 9AC reported here, as its inhibition would have hyperpolarized the cell and thereby reduced the firing frequency. However, blocking this voltage-dependent Cl^-^ current should have increases the R_input_ and decrease the rheobase, as it is open at rest. These effects were not observed here and thus, the decrease in firing frequency induced by 9AC is more likely to be induced by the effects of 9AC on Ca^2+^-dependent Cl^-^ channels and not on voltage-dependent Cl^-^ channels. In any case, more experiments will be necessary to ascertain whether this Cl^-^ current is present in mouse SCG neurons.

Although 9AC has been reported to activate an outwardly rectifying K^+^ conductance in rabbit smooth muscle cells (Toma et al., 1996), this effect appears when the membrane potential remains stable (in voltage-clamp experiments), at positive values above 30 mV. Hence, the reduction in repetitive firing produced here by 9AC was unlikely to be due to an increase in K^+^ conductances. The pharmacology of 9AC is complex, causing a voltage-dependent block of ANO2/TMEM16B in HEK cells (Cherian et al., 2015), although the main CaCC in SCG mice neurons is ANO1/TMEM16A. However, these earlier pharmacological studies were performed in an expression system and not on peripheral neurons, which might explain the differences in the behavior of the channels. Furthermore, we previously showed 9AC to be a more potent blocker (90% block) than niflumic (67% block) and flufenamic acid (50% block: Sánchez-Vives and Gallego, 1994; de Castro et al., 1997). This contrasts with data from CHO cells, in which niflumic acid blocks CaCCs more potently than other CaCC blockers (Liu et al., 2015), although again in experiments not performed on native tissues. Similarly, while new blockers of CaCCs may be more potent inhibitors of CaCCs in HEK 293 cells, such as T16A(inh)-A01 and CaCC(inh)-A01 (Bradley et al., 2014), these compounds produce concentration-dependent relaxation of rodent resistance arteries, equivalent to the vasorelaxation occurring when the transmembrane Cl^-^ gradient was abolished with an impermeant anion. Therefore, these compounds display poor selectivity for TMEM16A and for the inhibition of CaCCs (Boedtkjer et al., 2015) and thus, they were not used in our experiments.

As outlined in the Section “Introduction,” K^+^ currents are important in determining repetitive firing in sympathetic neurons. The I_M_ must be considered among these, and its enhancement by 9AC could explain the changes observed in repetitive firing. However, M channels can be directly and significantly inhibited by intracellular Ca^2+^ in patch-clamp experiments performed on dissociated rat SCG neurons, even within the basal range, and this inhibition is enhanced even by very small increments in [Ca^2+^]_i_ (Selyanko and Brown, 1996). The levels of [Ca^2+^]_i_ reached by depolarizations and repetitive firing in our experiments might be sufficient to inhibit I_M_, as evident in frog sympathetic cells (Tokimasa, 1985; Yu et al., 1994). Thus, this current could already be inactivated when we test the effects of 9AC. Furthermore, it is unclear whether 9AC affects the amplitude of I_M_. AHPs remain unchanged in the presence of 9AC, as occurs in guinea pig sympathetic neurons (Davies et al., 1999), suggesting that the I_K(Ca)_ is not affected either, and that the reduced firing frequency observed in the presence of 9AC must be due to another mechanism. 9AC does not affect K^+^ currents in smooth muscle cells from the rabbit portal vein (Hogg et al., 1994), although it inhibited K^+^ currents in pig atrial myocytes (Li et al., 2004), contributing to an increase in their firing rate as opposed to the effects observed here. Finally, the inactivation of Na^+^ channels does not seem to be responsible for the effects of 9AC in repetitive firing as the AP characteristics remain unchanged during prolonged firing. Indeed, 9AC had no effect on Na^+^ channels but rather it blocked a CaCC in cardiac ventricular cells (Váczi et al., 2015). Therefore, we believe that 9AC acts as a specific blocker of Ca^2+^-activated Cl^-^ channels in the experiments carried out here.

In this study, mouse SCG cells showed mixed firing patterns, approximately two thirds of which are Ph neurons and another third T neurons. Importantly, many of them showed repetitive firing, both Ph and T neurons, in contrast to previous studies in rat and mice where almost all SCG neurons fired phasically and lacked repetitive firing (Yarowsky and Weinreich, 1985; Adams and Harper, 1995; Wang and McKinnon, 1995; Jobling and Gibbins, 1999). The fact that adult mice of both sex may have been used, prompted us to compare the magnitude of CaCCs in male and female mice. Unexpectedly, the CaCC underlying the ADP was larger in males than in females, which may explain the differences in firing frequency between this and other studies. A methodological issue that could also account for the high degree of repetitive firing here is the fact that most of our recordings were performed with microelectrodes filled with high KCl concentrations. This might have increased the Cl^-^ concentration inside the cells and induced larger ADPs relative to other studies where lower KCl concentrations or even K-Acetate electrodes were used (Yarowsky and Weinreich, 1985; Wang and McKinnon, 1995; Jobling and Gibbins, 1999). However, as we demonstrated previously (de Castro et al., 1997), the ADP was still present when these neurons were impaled with K-Acetate electrodes. Furthermore, if the high KCl concentration in the electrodes induced a higher K^+^ concentration inside the cell, this might explain why the RMP in the cells studied is slightly more negative (5 mV) than previously reported (Jobling and Gibbins, 1999). The anesthetic employed here, pentobarbitone, as opposed to halothane (Jobling and Gibbins, 1999), may also have influenced the results. However, to our knowledge there is no specific and differential action of halothane or pentobarbitone on Ca^2+^-activated Cl^-^ channels that could provoke differences in the evoked ADPs and firing frequencies.

The anatomical distribution of neurons within the mammalian autonomous nervous system may be related to the activity of the targets, which indirectly modulate the activity of postganglionic neurons (McLachlan, 1987; Keast et al., 1993; Adams and Harper, 1995). However, the same targets and functions can be assumed for the SCG in both the rat and mouse. To explain differences between the two species, in the mouse Ph neurons may constitute a population of cells projecting to different targets to T neurons. Half of the sympathetic neurons from the mouse celiac ganglia fired tonically, while SCG and thoracic sympathetic neurons fired phasically (Jobling and Gibbins, 1999). Moreover, possible gender differences in the activation of the ADP in sympathetic neurons from rat and other species remain to be investigated.

Differences in the passive electrical properties of the sympathetic neurons have been reported across the prevertebral ganglia (Keast et al., 1993; Jobling and Gibbins, 1999) and these could account for the differences in their mode of discharge. For example, a sub-population of mouse cells with a higher R_input_ than others (and than rat cells) could explain the T response. Our data of R_input_ in mouse neurons agrees with the fact that it is inversely related to animal mass in sympathetic neurons (for a review, see: Adams and Harper, 1995), although we did not see significant differences in R_input_ between Ph and T cells. Similarly, other passive properties that could influence firing patterns must be taken into consideration, such as the V_threshold_ or the rheobase, although only the rheobase was different in both types of cells. While we did not measure the I_K(Ca)_, the characteristics of the AHP that follows a single AP are the same in Ph and T cells (see section “Results”), consistent with the idea that the AHP current is not responsible for the firing pattern (Wang and McKinnon, 1995; Jobling and Gibbins, 1999). Interestingly, the ADPs in Ph and T neurons display no differences. Thus, we conclude that while CaCCs do not define the firing behavior of a ganglion cell (Ph or T), paradoxically they contribute to enhancing the firing rate cell-by-cell (see below). In fact, the firing frequency was affected by inhibition of CaCC by 9AC, especially when higher frequencies were reached, although the differences were significant for all amplitudes of the depolarizing pulses tested. Discharge firing was affected equally in cells with a clear ADP post-train as in those with AHPs, or when Ph and T neurons were compared. The instantaneous frequency was also significantly reduced in the presence of 9AC, supporting the importance of CaCCs for repetitive firing in SCG cells. Together, these data show that CaCC blockade reduces the firing frequency of these neurons.

Although cells with no repetitive discharges cannot be considered in the same way (see below), their responses to long depolarizations remained unchanged in the presence of 9AC. The fact that the same behavior was observed in the presence and absence of 9AC when a single spike is generated after long depolarizations, and that the latency of the first spike burst did not significantly change when 9AC was used (see section “Results”), suggests that CaCC activation is not necessary to generate the first AP but rather, it is required for repetitive firing. Indeed, these cells do not elicit a spontaneous spike during the ADPs in the rat (Sánchez-Vives and Gallego, 1993, 1994) or mouse (de Castro et al., 1997), which could be due to a large decrease in the input resistance of the cells during ADP when both CaCC and I_K,Ca_ are activated (Sánchez-Vives and Gallego, 1993).

Of all the electrical properties tested here, we conclude that 9AC affects mainly the ADP generated after a train of high frequency spikes (**Table 2**). Neither the amplitude nor the duration of the spike changed when CaCCs were blocked with 9AC, suggesting that this current does not play a substantial part in generating single APs. This is in agreement with data obtained from rabbit parasympathetic ganglia, where modifications of both external and internal Cl^-^ concentrations do not significantly alter the properties of individual APs in cells with a Ca^2+^-activated Cl^-^ current (Nishimura, 1995), as demonstrated more recently in cardiac ventricular cells (Váczi et al., 2015). The firing of excitable cells is not only affected by the characteristics of the AP but also, by the R_input_ and rheobase. In our case, the R_input_, rheobase and V_threshold_ did not change when the CaCCs were blocked. Thus, the dramatic effect of 9AC on firing behavior suggests that changes in firing frequency are almost entirely due to the blockade of CaCCs in sympathetic ganglion cells.

We observed a slight hyperpolarization of the RMP (≈4 mV) in neurons treated with 9AC, possibly due to a mild increase in the resting intracellular Ca^2+^ that might have partially activated CaCCs. This might be due to electrode impalement, and that subsequent application of 9AC to block CaCCs reverses this effect. Alternatively, 9AC could have blocked a voltage-dependent Cl^-^ inward current (Sacchi et al., 1999). However, either of these two possibilities should have increased the R_input_ and decreased the rheobase, which was not the case. Nevertheless, the effects of 9AC on firing frequency and instantaneous frequency were identical in neurons that were slightly hyperpolarized and in neurons in which the RMP remained stable in 9AC.

We also tested the possibility that the CaCCs could be involved in the synaptic transmission of sympathetic cells. CaCC blockade with 9AC did not alter synaptic transmission and the EPSPs generated in the refractory period by stimulating the preganglionic branch were not affected by 9AC. Hence, CaCCs do not appear to be important for synaptic transmission, at least at low frequency stimulation (1 Hz), similar to the frequency of spontaneous discharge observed in anesthetized mice *in vivo* (Ivanov and Purves, 1989; McLachlan et al., 1998). CaCCs could be activated by a repetitive stimulus at a higher frequency, when perhaps the intracytoplasmic Ca^2+^ levels increase enough to activate the current. We concluded that the Ca^2+^-dependent Cl^-^ channels responsible for ADPs located preferentially to the terminal dendrites (de Castro et al., 1997). Although it was thought that increasing Ca^2+^ levels in dendrites only occurs when neurons fire at high frequencies (Markram et al., 1995), the development of two-photon Ca^2+^ imaging allowed Ca^2+^ signaling in neuronal dendrites to be monitored in more detail (Denk et al., 1995), showing that Ca^2+^ transients can be observed as a consequence of single AP firing in neurons (Goldberg et al., 2003). Hence, even at physiological frequencies of AP firing, the Ca^2+^-dependent Cl^-^ channels underlying the ADP could modulate the frequency of sympathetic neuron firing.

A post-tetanic depolarization produced by activation of CaCCs in parasympathetic neurons has been proposed to cause a slow EPSP (Nishimura, 1995). The same function could be attributed to this current in sympathetic neurons, stronger activation of CaCCs at high frequencies depolarizing the cell until the firing threshold is again reached, and its blockade would prolong the predominant effect of I_K(Ca)_, delaying the generation of the next spike. An ADP following an AP and its AHP has been recorded in about 50% of the rat coeliac-superior mesenteric ganglion cells after both single and repetitive firing, and it is modulated by substance P (Konishi et al., 1992). Interestingly, the ADP was attributed to a deactivation of some I_K(Ca)_ channels, although the possibility of other ions being involved in that phenomenon was considered. The biophysical properties of CaCC make this current a candidate for this effect and thus, the relative magnitude of CaCCs and I_K(Ca)_ seems to be crucial to establish the firing frequency of these neurons.

It is well established that the balance between excitation and inhibition is of paramount importance for the correct function of the nervous system. Indeed, many neurodevelopmental brain disorders may arise from imbalances in excitatory and inhibitory brain circuitry, including schizophrenia and autism (O’Donnell et al., 2017). Na^+^, K^+^, and Ca^2+^ ion channels are the proteins studied most extensively in the shaping of neuron excitability, particularly since the pharmacology of Cl^-^ channels has hindered the better understanding of their physiological importance. However, as discussed here 9AC can be considered as a specific blocker of Ca^2+^-dependent Cl^-^ channels in our model. The physiological roles of Cl^-^ channels are impressive, ranging from the regulation of cell volume, transepithelial transport, and even electrical excitability. A role of Cl^-^ channels in volume was suggested in normal neurons (Alvarez-Leefmans et al., 1988; Hussy, 1992) and in rat axotomized sympathetic neurons, where they could help to restore normal volume after osmotic changes induced by axonal lesion (Sánchez-Vives and Gallego, 1993). The loss of distinct Cl^-^ channels leads to cystic fibrosis and Bartter’s syndrome, increased muscle excitability in myotonia congenita and it may result in blindness in mice (Jentsch et al., 2002). Correct Cl^-^ ion channel function is crucial to balance the excitation and inhibition of several neurons, as shown recently in olivary neurons in which a Ca^2+^-activated Cl^-^ channel contributes to the repolarization of an ADP and therefore increases the firing rate, thereby promoting cerebellar motor learning (Zhang et al., 2017). In central neurons of the dorsal motor nucleus of the vagus, axotomy disrupts intracellular Cl^-^ regulation, causing a shift from an inhibitory to an excitatory response to GABA, which might contribute to excitotoxicity in injured neurons (Nabekura et al., 2002). We have evaluated the effect of axotomy on ion channel physiology in sympathetic neurons, showing an increase in Ca^2+^-activated Cl^-^ channel expression and larger ADPs (Sánchez-Vives and Gallego, 1994).

As outlined above, the CaCC underlying the ADP in mouse SCG sympathetic neurons was larger in males than in females. Furthermore, ANO1 is expressed in mouse sympathetic SCGs, which is again stronger in male than in female ganglia. Although gender differences have been appreciated in GABA-mediated excitation during development, these differences are explained by the distinct expression of K^+^-Cl^-^ or Na^+^-K^+^-Cl^-^ cotransporters in male and female animals (Galanopoulou, 2005; Nuñez and McCarthy, 2007), and not by differences in the expression of Cl^-^ channels, particularly in the PNS. However, this differential expression of Cl^-^ ion channels in male and female sympathetic neurons contributes to the firing frequency, which may explain the gender differences in the activation of sympathetic tone observed recently in mice (Bruder-Nascimento et al., 2017).

All these observations indicate that the balance between the activity of Cl^-^ channels and other ion channels contributes to establish the electrical activity of the cell in its physiological context and alterations in this equilibrium may challenge homeostasis. In our model, mouse sympathetic ganglion neurons, increased Ca^2+^-activated Cl^-^ channel activity is evident relative to that of Ca^2+^-activated K^+^ channels and in comparison with other species (or in male vs. female mice), producing larger ADPs that lead to a higher firing frequency.

## Author Contributions

JM-P and FdC designed the study. JM-P, SS, ET, and FdC collected and analyzed the data. JM-P, SS, ET, AN, and FdC wrote the paper and edited the manuscript.

## Conflict of Interest Statement

The authors declare that the research was conducted in the absence of any commercial or financial relationships that could be construed as a potential conflict of interest.
