# Supplementary material for: A Calcium-Dependent Chloride Current Increases Repetitive Firing in Mouse Sympathetic Neurons
Source: Front Physiol. 2018 May 14;9:508. doi: 10.3389/fphys.2018.00508 (PMC5960682; doi:10.3389/fphys.2018.00508)
Supplement: Supplementary file 1 [file Presentation_1.PDF]

Supplemental material

Recording time does not affect the electrical properties of mouse sympathetic neurons.

Since the effects of 9AC were maximal 2-4 minutes after its application, it is possible that the cell becomes damaged during the period of intracellular recording. To exclude this possibility we studied the electrical properties in a group of neurons (n = 6) immediately after impaling the cell with the microelectrode and 3-6 minutes later (time necessary for the complete recording and the change of solutions). All the electrophysiological parameters measured were the same in both, including those related to the AHP post-spike (amplitude and half-duration) and the amplitude and duration of ADP post-train (data not shown).

We tested whether the firing frequency changed during impalement: as can be seen in supplemental Fig. 1, no significant differences were found either in the pattern and frequency of firing (Supplemental Fig. 1A and B) or in the instantaneous frequency (Supplemental Fig. 1C). No differences were observed when only cells that hyperpolarized ≥ 3 mV were studied after 3-6 minutes of impaling in control, either for the electrophysiological parameters or for repetitive firing (data not shown).

Similarly, in five of these cells, no changes in the EPSP amplitude were detected when the same cell was repeatedly tested in control solution with intervals of 3-6 minutes or longer (data not shown).

Therefore, the effects observed during 9AC superfusion were not due to cell damage produced by the impalement.

Figure S1

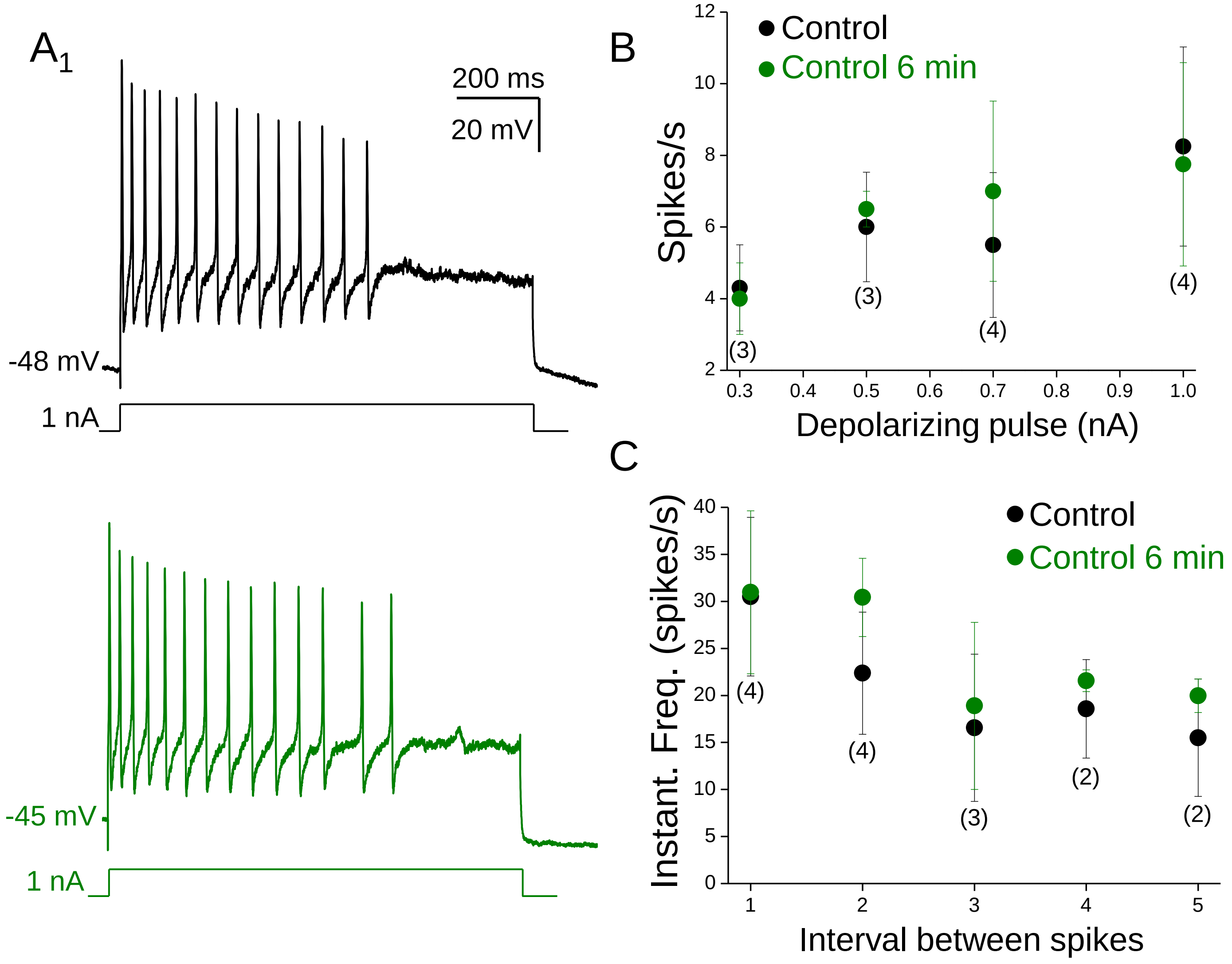

Fig. S1: Impaling time does not affect the electrical properties of sympathetic neurons. *A*: Repetitive firing in response to a long (1 s) 1.0 nA depolarizing pulse (upper black trace) does not change after 6 minutes of impaling (lower green trace), both recordings are in control solution. The resting membrane potential of the cell is indicated at the beginning of each recording. Neither firing frequency at different depolarizing pulses (*B*) nor instant frequency of spikes measured in the first five intervals between consecutive spikes (*C*) change between the first recordings in control solution (black circles) and after 3-6 minutes of impaling (green circles) in the same solution. In brackets is the number of cases studied in each case. None of the electrophysiological parameters changed with this maneuver.
